# Mixed Micromax and hematite-based fly ash geopolymer for heavy-weight well cementing

**DOI:** 10.1038/s41598-023-36010-w

**Published:** 2023-05-29

**Authors:** Ahmed Abdelaal, Salaheldin Elkatatny

**Affiliations:** Department of Petroleum Engineering, College of Petroleum and Geosciences, King Fahd University of Petroleum and Minerals, 31261 Dhahran, Saudi Arabia

**Keywords:** Engineering, Chemical engineering, Civil engineering

## Abstract

Ordinary Portland cement (OPC) has introduced different environmental and technical issues. Researchers tried either adding new materials to cement or developing alternatives for both technical and environmental challenges. Hematite as a weighting agent is used to increase cement slurry density. Heavy particles sedimentation in cement and geopolymer slurries is a serious issue which creates heterogenous properties along the cemented section. This work presents a new class of geopolymers using both hematite and Micromax as weighting materials for high density well cementing applications. The first system used only hematite while the other system used both hematite and Micromax. The main goal behind using Micromax with hematite is to check the possibility of eliminating the sedimentation issue associated with hematite in geopolymers. Moreover, the effects of adding Micromax on different FFA geopolymer properties were also evaluated. Different mixtures of retarder, retarder intensifier and superplasticizer were introduced to increase the thickening times of the developed geopolymer systems. The results showed that adding Micromax to hematite decreased the average density variation from 12.5% to almost 3.9%. Micromax addition reduced plastic viscosity by 44.5% and fluid loss by 10.5%. Both systems had a close performance in terms of strength, elastic properties, and permeability. The thickening time was 390 min for the hematite system and 300 min for the mixed system using the proposed additives mixtures.

## Introduction

OPC introduces some technical and environmental challenges such as high greenhouse gases emissions and consumes massive energy during its production. These concerns persuaded the researchers to search for alternative materials to overcome the technical challenges and to provide eco-friendly cement systems. It is obvious from the literature that some researchers flew in the direction of adding new materials to OPC to enhance its properties^1–7^. Other researchers tried to find new alternatives to OPC which can be more eco-friendly and can overcome the technical drawbacks of OPC such as geopolymers^8–13^.

Currently, a cost-effective and ecofriendly substance has emerged that has qualities comparable to OPC; this substance is termed as geopolymer. Its raw materials are of geological origin and geopolymer formation continues via inorganic polymerization and condensation, so they are also called geological polymers^14^. Prof. J. Davidovits coined the term geopolymer in 1978, describing it as a green cementitious substance that is free of cement. As it contains a 3D structure of cross-linked polysialate chains, these were previously regarded a particular case of soil cement and referred to as geocements^15^. Geopolymers can be formed from natural substances and/or waste materials as source materials activated by alkali or acids^16^. Geopolymer production is cleaner, and the source materials do not consume that much energy as compared to OPC^17–20^. The Si and Al in the aluminosilicate substances dissolve after contacting the alkali solutions to from monomers and oligomers going through polycondensation to form a 3D structure, called, polysialate, polysialte-siloxo, and polysialate-disiloxo^21,22^.

Cement slurry is designed depending on the existing wellbore conditions of pressure and temperature and cement job type. Most research has been conducted on not just searching for an alternative to OPC due to its environmental concerns during production but most significantly on confirming if the drawbacks of OPC can be mitigated by geopolymers. Four main research topics were investigated in the literature: applications in harsh environments, application in P&A, compatibility with mud, and temperature effects^10,23–28^.

In the petroleum industry, high pressure high temperature (HPHT) wells drilling represents a new frontier. The exploration and production industry looks for new resources to meet the rising demand of energy across the world and some of these resources are buried deeper in the earth crust. Over half of known petroleum reserves in the US are located below 14,000 feet subsea. HPHT conditions will be encountered as we dig further into the strata. As a result, businesses are obligated to fulfill or exceed a wide range of technical constraints as well as environmental, health, and safety regulations^29^. Special challenges are associated with cementing operation in HPHT wells owing to the physical and chemical changes under high pressures and temperatures. These harsh conditions can introduce challenges not only while placement but also after cement setting^30,31^. Decreasing water content is the easiest way to increase the slurry density^32^. However, the American petroleum institute (API) advises a water-to-cement ratio of 0.44 for primary cementing (API, 2019). Water content reduction has challenges such as attaining sufficient fluid loss control, appropriate rheology, and no settling of the solid particles^33^. Slurry bridging occurs due to uncontrollable fluid losses. Solid particle settling results in nonuniform compressive strength and bonding along the length of cemented section^34^.

Therefore, using weighting materials is required to achieve a higher density. Using weighting materials in geopolymers research area for the oil and gas industry is relatively new. These attempts used barite to study its effects on compressive strength and thickening time. Kanesan *et al*.^35^ measured the thickening time of the barite-based FA geopolymer at 140℉ and 2000 psi using a combination of 8 M NaOH solution and Na_2_SiO_3_. The authors mentioned that retarder effect was major in low and medium density slurries, but it had a minor effect on high-density slurry thickening time. Salehi *et al*.^36^ investigated the impact of adding 15% BWOC barite on compressive strength of FA geopolymers. Barite improved strength up to 7 days of curing and could not enhance the strength after 7 days. It is clear from the literature that few trials were performed using barite with geopolymers for well cementing applications. The big particles of weighting agents may cause particles sedimentation^37^. The sedimentation problem in slurries creates a heterogenous cement column which is not favorable for strength and zonal isolation^34^. Abdelaal *et al*.^38^ introduced a new heavy weight fly ash geopolymer formulation which used hematite as a weighting agent. Although this formulation provided a geopolymer system with accepted cement properties, sedimentation issue was not investigated in this study.

Geopolymer technology is still evolving in the petroleum industry, and it has not seen a full-scale deployment in well cementing. Providing of moderate to high density geopolymer cement systems using various weighting materials is rarely investigated in the literature. This study introduces the first usage of Micromax in geopolymer slurries for oil well cementing. The first goal in this work was generating flowable heavy weight geopolymer formulations with appropriate rheology and appropriate thickening time. The second goal was to solve the sedimentation issue associated with the hematite based geopolymer by introducing a mixture of Micromax and hematite. The sedimentation of the weighting materials was investigated by the API method. In addition, various properties such as rheological properties, filtrate loss, strength (compressive and tensile), permeability, elastic properties were also evaluated for hematite based and mixed Micromax and hematite geopolymer systems.

## Materials and methods

### Materials

**Figure 1:**
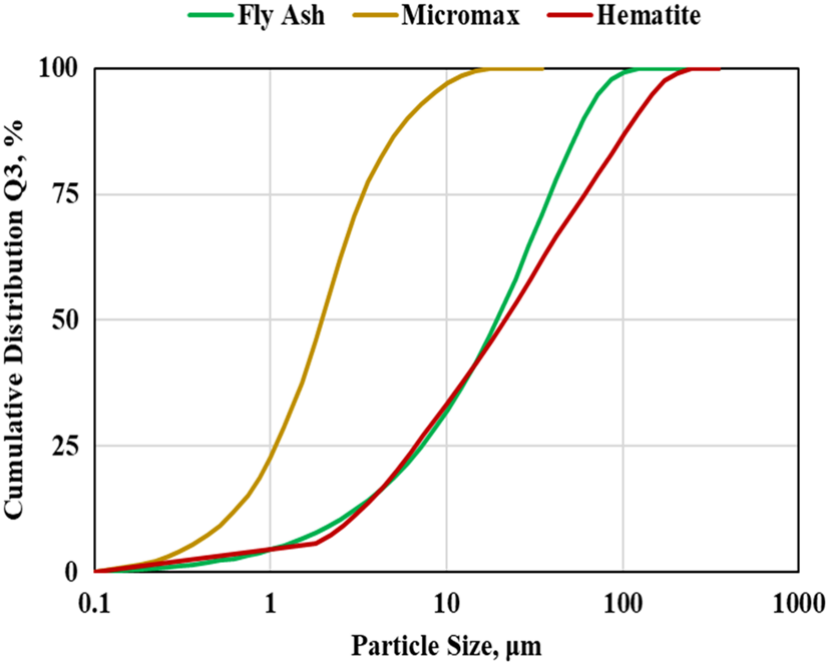
PSD results of the Class F FA, hematite and Micromax.

The materials used in this work were Class F fly ash as a source material, hematite and Micromax as weighting materials, 4M NaOH solution as an activator in addition to other additives utilized to enhance geopolymer characteristics and make it flowable under wellbore conditions. The additives comprised retarders, retarder intensifiers, deformers, and superplasticizers. The specific gravities (SG) of the used materials are listed in Table 1. The particle size distributions (PSD) were obtained using the laser diffraction particle size analyzer as shown in Fig. 1 and summarized in Table 1. The results showed that the median size of FFA, hematite and Micromax were 19.35, 21.54 and 1.98 µm respectively.

**Table 1:** The SG and PSD results of FFA, hematite and Micromax.

	FFA	Hematite	Micromax
SG	2.25	5.05	4.68
D_10_ (µm)	2.38	2.77	0.55
D_50_ (µm)	19.35	21.54	1.98
D_90_ (µm)	60.00	117.38	6.05

**Figure 2:**
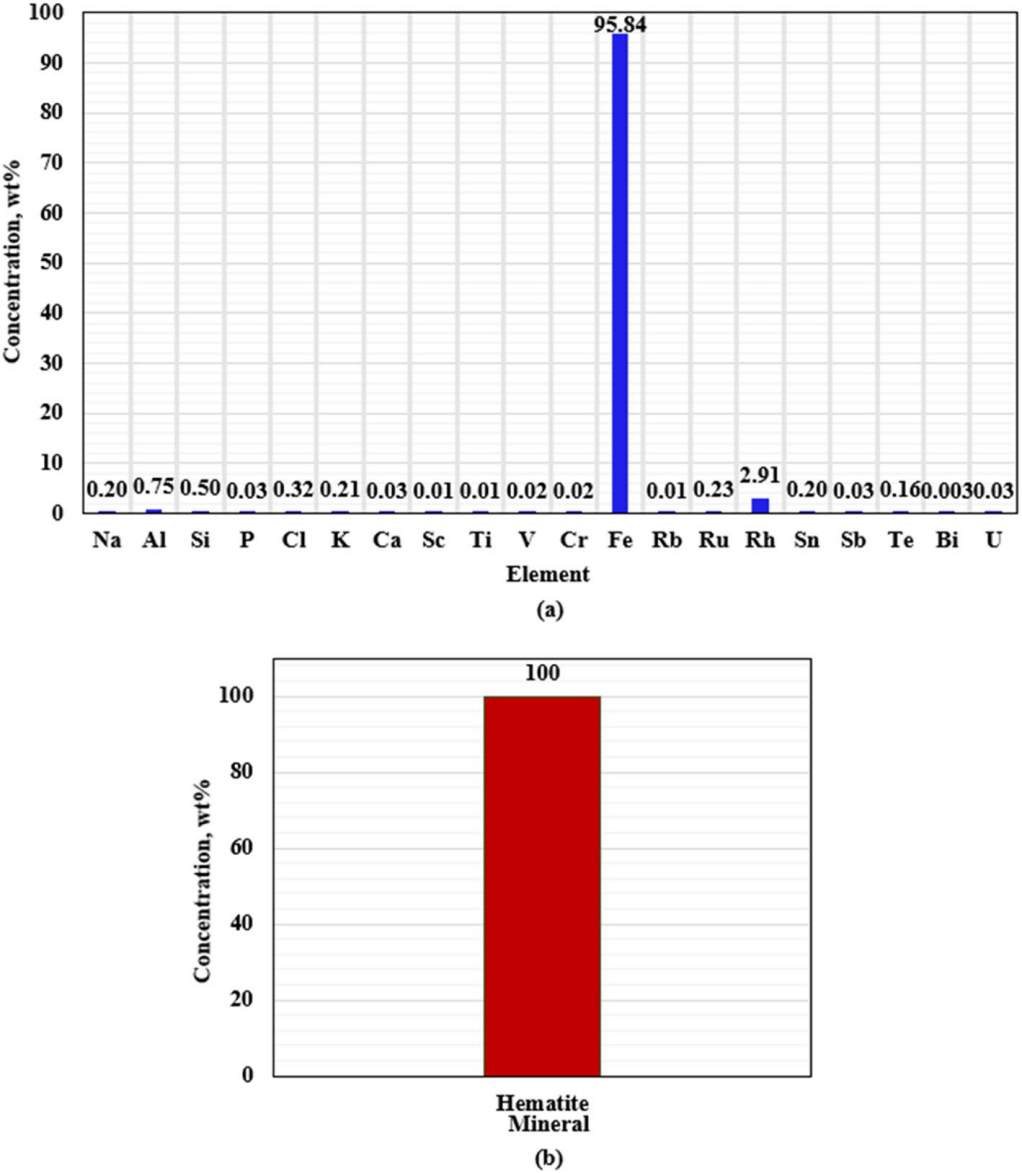
XRF elemental composition of hematite (**a**) and the XRD of the sample (**b**).

**Figure 3:**
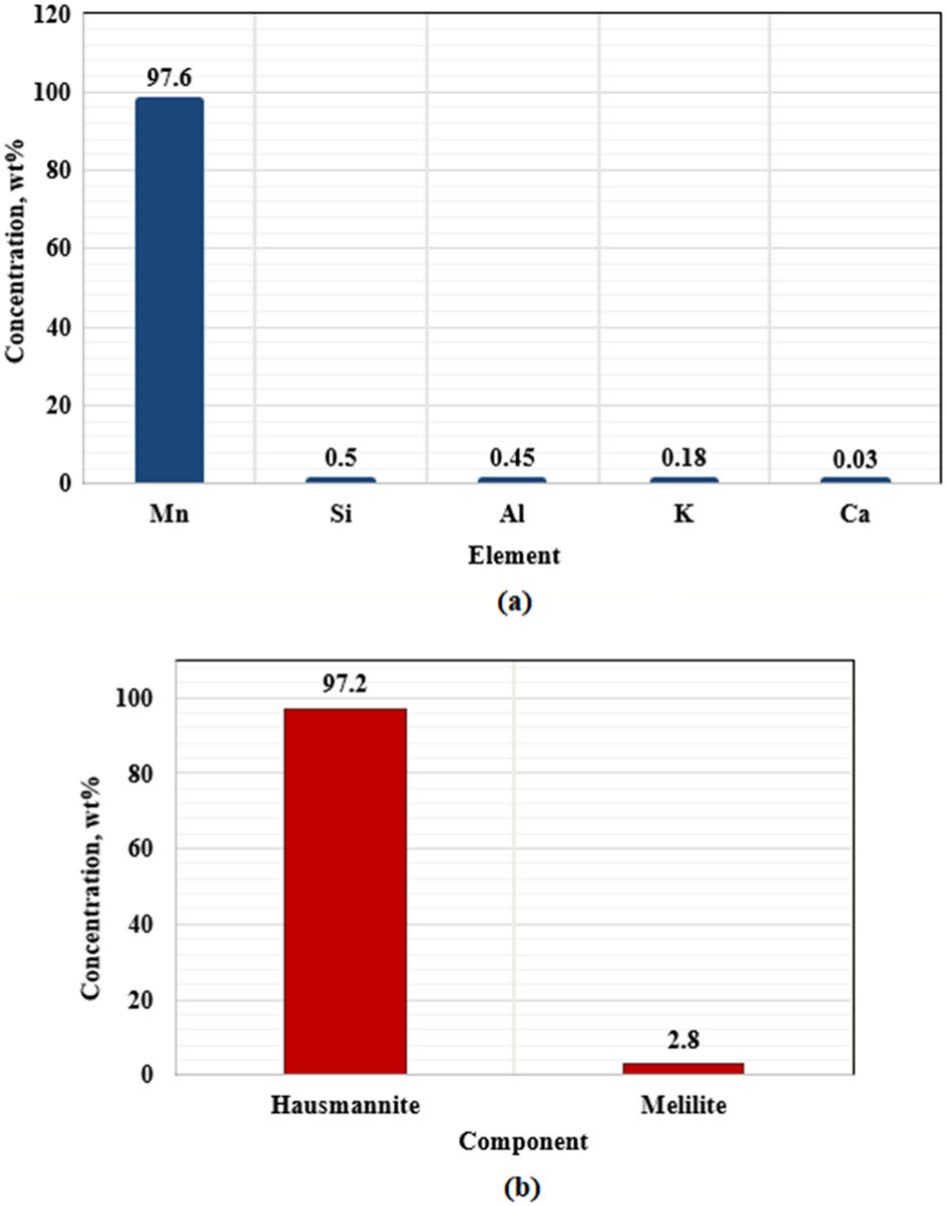
XRF elemental composition of Micromax (**a**) and the XRD of Micromax (**b**).

**Figure 4:**
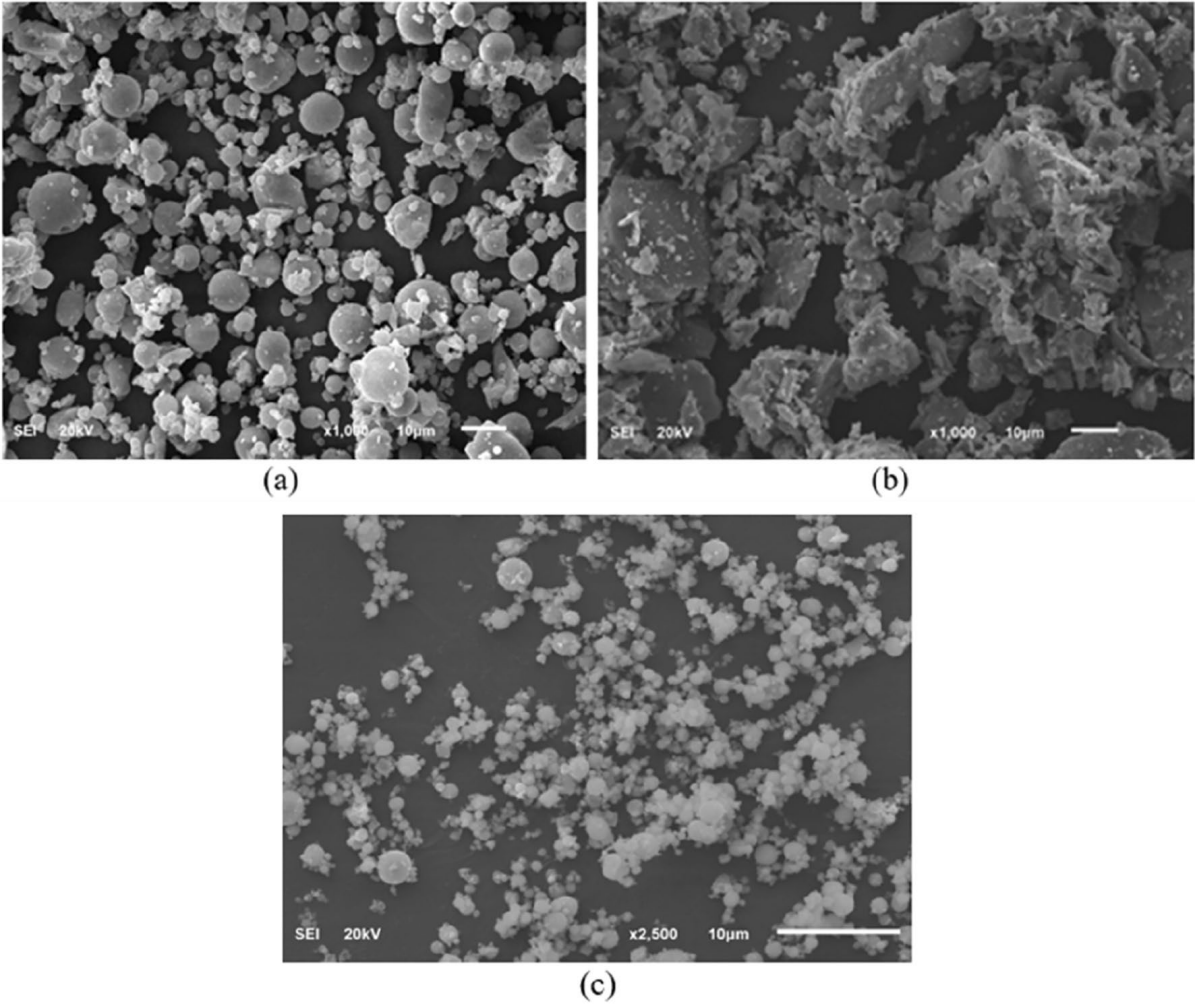
SEM images for FFA (**a**), hematite (**b**) and Micromax (**c**).

Figures 2 and 3 show the elemental composition of hematite and Micromax, respectively used in this work obtained by X-ray fluorescence (XRF). XRF results confirm that hematite has a high iron amount (around 95%) and X-ray diffraction (XRD) showed that it contained 100% hematite. XRF also showed that FFA had considerable amounts of silica (SiO_2_) and alumina (Al_2_O_3_), as listed in Table 2, which play a vital role in geopolymer formation. The scanning electron microscope (SEM) results, as shown in Fig. 4, show that FFA and Micromax particles are more like spheres and hematite particles possess irregular shapes.

**Table 2:** XRF results of FFA used in this study.

Oxide	Percentage (%)
SiO_2_	55.92
Al_2_O_3_	29.54
CaO	5.49
Fe_2_O_3_	4.93
TiO_2_	1.91
K_2_O	1.66
SO_3_	0.39
Others	0.17

### Methods

**Figure 5:**
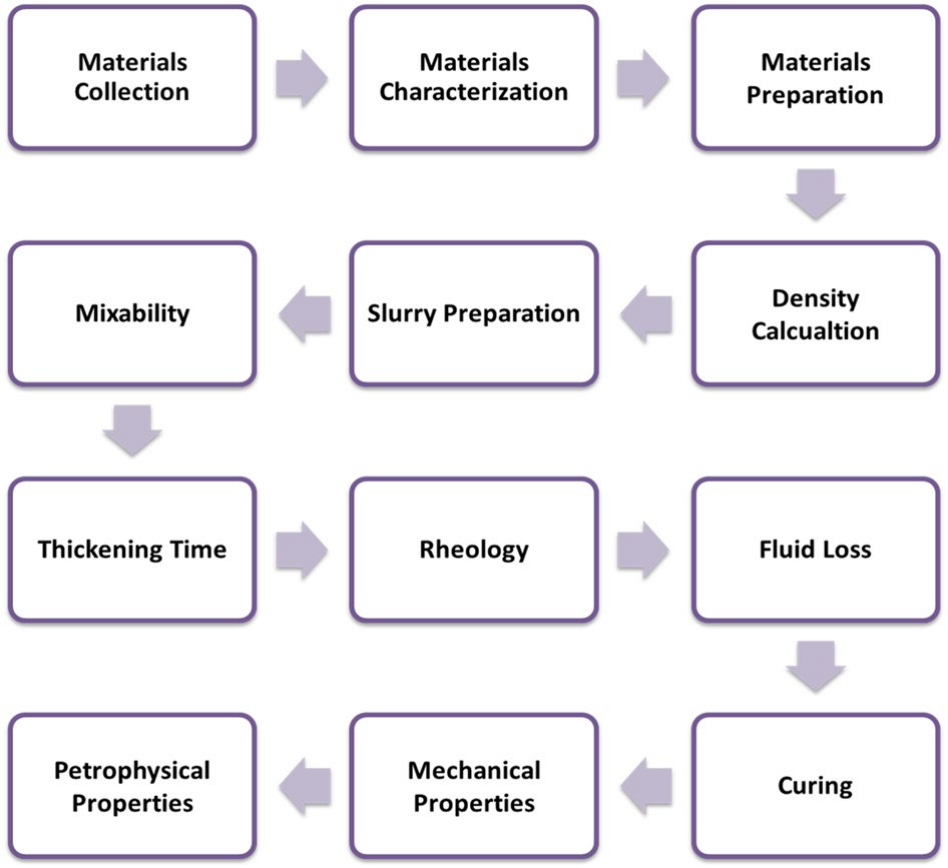
The summary of methods in this study.

This part discusses the methods followed to perform this work. It began with collection, characterization, and preparation of materials. Then, the slurry ingredients were determined based on mixability, pumpability and rheology. Afterwards, the hardened geopolymer samples were evaluated for mechanical and elastic properties. Figure 5 summarizes the methodology followed in this study.

#### Materials preparation

Sodium hydroxide solution was prepared by dissolving its pellets in distilled water using a magnetic stirrer. Then, it was left to cool down to atmospheric temperature at least for 24 h before experiment. The specific gravity of the powders was estimated to determine the required amounts to reach the required density. The FFA was sieved below 104 μm and then was confirmed by PSD. Two different processes (wet and dry) can be followed to prepare geopolymer slurry^36^. In the wet process, a superplasticizer and/or retarder are mixed with activation solution for 2 min at high shear rate (12,000 RPM) using constant speed mixer. A binder such as FFA is then added and mixed with the premixed solution at a high shear rate for another 2 min. To create a sample using the dry process, sodium hydroxide powder is combined with the desired quantity of sodium silicate and allowed to cool for a period of two hours. Next, the desired amount of water is mixed with a suitable plasticizer, followed by the addition of fly ash. Finally, a mixture of water solution and alkali solution are mixed.

#### Slurry design

Different trials were performed to obtain an initial flowable slurry formulation that can be easily mixed. Different parameters were tested such as NaOH molarity (4, 6, 8 and 10M), liquid to binder ratio (0.5 to 0.8), and weighting material amount. In addition, several additives with different concentrations were tested such as retarders, retarder intensifiers and superplasticizers to enhance the slurry workability. The mix design started with testing mixability, thickening time and rheology. Then, testing went through strength, elastic and petrophysical properties and sedimentation evaluation.

#### Rheology, fluid loss, and thickening time

After mixing, an atmospheric consistometer is used for conditioning the geopolymer slurries at 195℉ and 150 RPM for 30 min. The OFITE 900 viscometer was used to evaluate rheology at average temperature of 192℉ and atmospheric pressure. The HPHT filter press was used to measure the fluid loss. The thickening time was evaluated at 195℉ and atmospheric pressure using an atmospheric consistometer to know how long slurry would remain pumpable.

#### Slurry stability testing

The geopolymer slurries were conditioned to simulate dynamic placement in wellbores and were then left static to determine if free fluid separates from the slurry. After curing, the cylindrical samples were cut into 3 sections (bottom, middle and top). Then, the sedimentation test procedure was followed by measuring the weight of each section in water and air. The density of each section was calculated by dividing the weight in air by the weight in water according to Archimedes Principal. The density difference of each section was calculated using Eq. 1. The acceptable density difference varies with the application type.

1 $$\% \,Density \,Difference=\frac{Cement\, Segment\, Density - Cement\, Slurry\, Density}{Cement\, Slurry\, Density}*100$$

#### Mechanical and petrophysical properties

Mechanical properties evaluation included unconfined compressive strength (UCS), tensile strength, dynamic elastic properties (Young’s modulus (YM) and Poisson’s ratio (PR)) and permeability was the evaluated petrophysical property. After conditioning, the slurry was poured into cubic (2 in. in length) and cylindrical (1.5 in. in diameter and 4 in. in length) molds and then were placed in an HPHT curing chamber at 292℉ and 3000 psi for 24 h. The Brazilian test procedure was followed to measure the tensile strength^39^. The UCS was evaluated using the scratch test. The scratch test is designed to regulate and monitor the continual shearing action caused by the movement of a diamond cutter on the sample surface. The force operating on the cutter generates a continuous profile of rock strength along the sample. The YM and PR were then determined by getting the sonic velocities. The ultrasonic test measures how long it takes a pressure wave to travel between two probes. The compression and flexural machine was utilized for tensile strength analysis, while scratch testing machine with sonic mode was used for elastic properties and UCS. Nitrogen gas was used for measuring gas permeability at room temperature and a confining pressure of 1,000 psi. The gas permeability was measured at different pressures and a graph was constructed between gas permeability versus 1/p_mean_. Then, a straight line was extrapolated to obtain the intercept which refers to the liquid permeability.

## Results and discussion

### Formulation design

The hematite-based system mix design is listed in Table 3. Although, this geopolymer system possessed acceptable properties, it showed density variation along the cylindrical samples due to hematite sedimentation. Replacing part of hematite with another weighting agent such as Micromax was investigated to tackle the sedimentation issue associated with hematite. The proposed mixture of retarder and superplasticizer that improved the thickening time of the hematite-based system did not have the same performance with the mixed system. As a result, different mixtures of additives were tested until obtaining a sufficient thickening time of around 5 h using a retarder intensifier with a retarder and a superplasticizer. The mix design was adjusted to replace 50% of hematite with Micromax. The proposed additives mixture allowed using only 0.5 liquid to binder ratio to have a flowable slurry with a reasonable rheology. The proposed formulations design for both systems are shown in Table 3 and expressed as by weight of binder (BWOB).

**Table 3:** The mix design used in this work.

Component	SG	Hematite System (BWOB, %)	Mixed System (BWOB, %)
FFA	2.25	100	100
Hematite	5.05	80	30
Micromax	4.68	–	30
Defoamer	1.1	0.0164	0.0164
Superplasticizer	1.21	5	5
Retarder	1.22	5	3
Retarder Intensifier	1.76	–	2
4 M NaOH	1.15	56	50

### Thickening time

**Figure 6:**
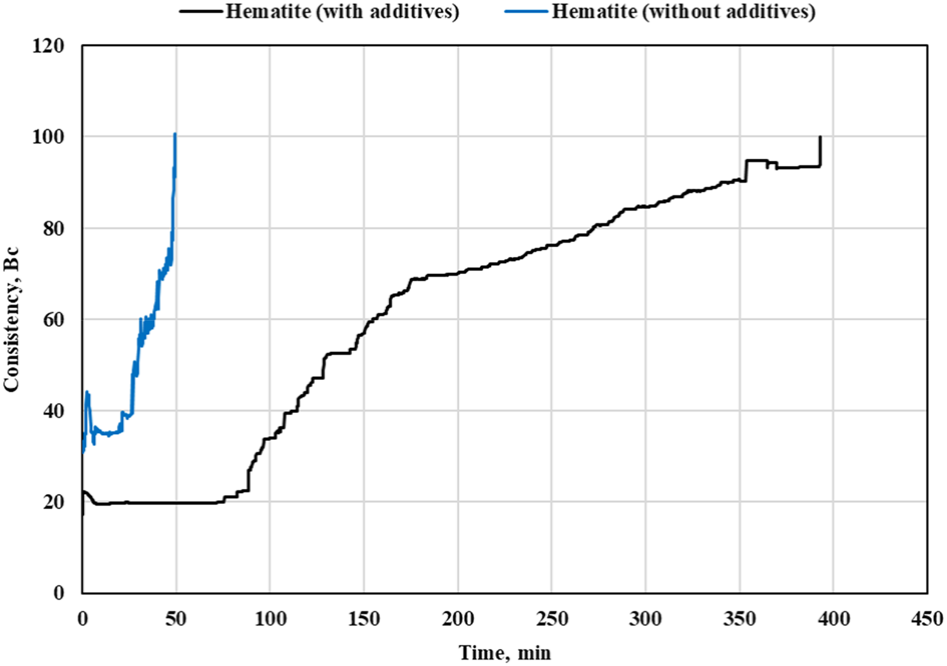
Thickening time chart for the hematite geopolymer system.

**Figure 7:**
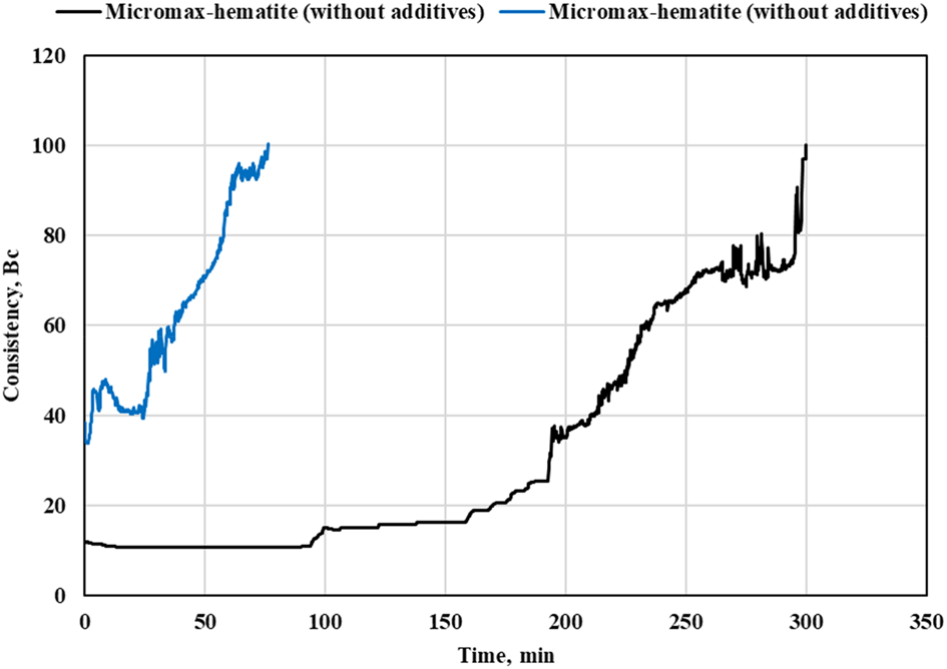
Thickening time chart for the mixed hematite-Micromax geopolymer system.

When the slurry was prepared by mixing FFA, hematite and NaOH solution, the thickening time at BHCT of 195℉ was around 50 min. This thickening time was too short to place the cement behind the casing. Different types and mixtures of retarders, dispersants, superplasticizers were tested to prolong the pumpability of geopolymer slurries. Using superplasticizer (5% BWOB) slightly increased the thickening time and the same for the retarder. When both were used together (5% BWOB each), the thickening time increased from 50 to 390 min. For the mixed Micromax-hematite system, the developed additives mixture increased thickening time to only 180 min. Consequently, different mixtures were tested to increase the thickening time of the mixed system after adding Micromax. Another mixture of a retarder (5% BWOB), superplasticizer (3% BWOB) and a retarder intensifier (2% BWOB) increased the thickening time from 76 to 300 min at BHCT of 195℉. The developed geopolymer slurries, as shown in Figs. 6 and 7, did not have a right angle set unlike most of cement slurries. Thickening time charts for both systems are shown in Figs. 6 and 7.

### Rheological properties

**Figure 8:**
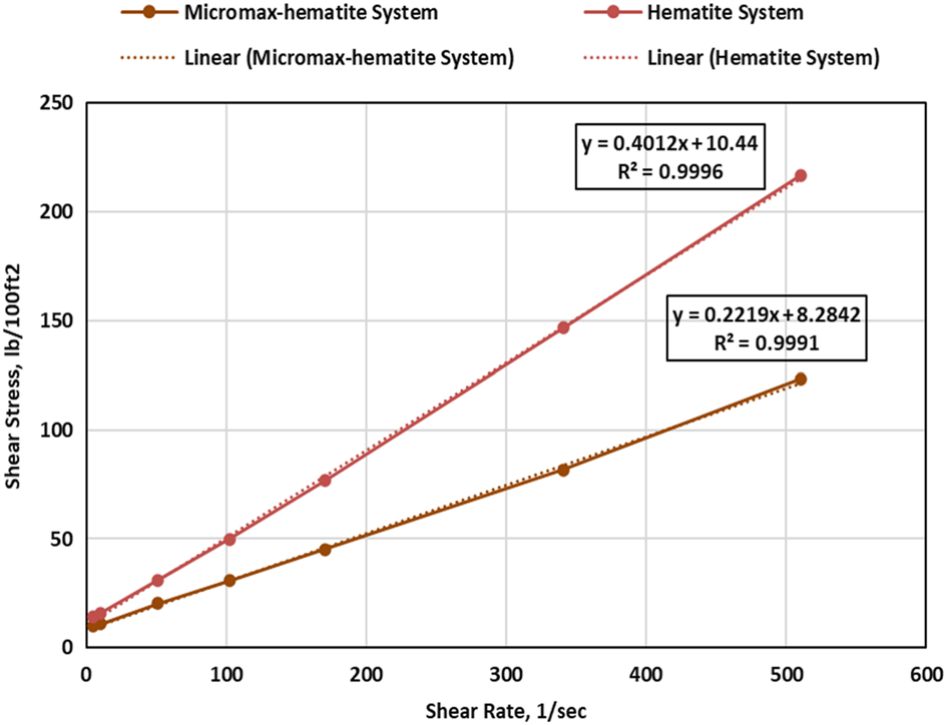
The shear stress vs. shear rate of the developed geopolymer slurries.

**Figure 9:**
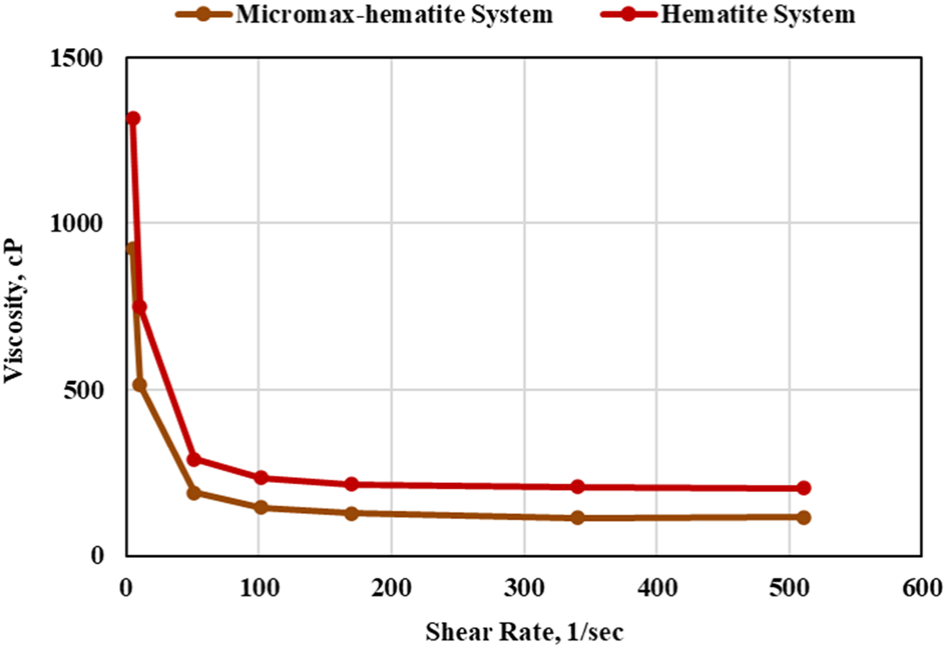
The viscosity vs. shear rate of the developed geopolymer slurries.

Evaluation of the rheology of cement slurries is important because it affects slurry mixing, pumping, and mud displacement. After conditioning for 30 min at 195℉, the rheology measurements were conducted using the viscometer. The shear stress and viscosity against shear rate are shown in Figs. 8 and 9, respectively. The Bingham Plastic model offered the best fit for the rheology of both systems with R^2^ of 0.999. Adding Micromax to hematite decreased the plastic viscosity by 44.8%. Micromax particles are more like spheres, as shown in Fig. 4, that minimize the friction of particles interaction and result in a low plastic viscosity.

Yield point determines the force that should be overcome to initiate the flow and reflects the slurry carrying capacity under dynamic condition. Therefore, the yield point should not be either that high or that low. The carrying capacity of slurry under static condition is determined by gel strength, and the gel's growth aids in preventing gas migration. Both systems had a close performance in terms of yield point and the hematite-based system was more in terms of gel strength (GS) as presented in Table 4. Both systems had shear thinning behavior as the apparent viscosity reduced by increasing the shear rate as shown by Fig. 9.

**Table 4:** The rheological properties of the developed slurries.

Property	Unit	Hematite system	Hematite-Micromax system
PV	cP	192.49	106.24
YP	lb/100 ft^2^	10.5	8.3
10 s GS	lb/100 ft^2^	16.0	11.3
10 min GS	lb/100 ft^2^	171.0	130.0

### Fluid loss

It is advisable to minimize the slurry fluid loss into permeable zones to decrease hydration of water-sensitive shales and prevent increasing slurry viscosity while placement. Managed fluid loss also decreases annular bridges that can act like a packer and remove hydrostatic pressure holding back potentially over-pressure zones. In addition, it reduces cement dehydration during pumping into abandoned perforated interval allowing for plugging longer interval of perforations in a single job. Volume reduction occurs while cement makes transition from a liquid slurry to a solid cement. If the fluid loss is high, much larger volume reductions are thought to be possible that may facilitate gas migration^40^. The hematite-based slurry had a fluid loss of 38 mL/30 min while the mixed hematite and Micromax slurry had a fluid loss of 34 mL/30 min. The developed slurries fall within the acceptable fluid loss ranges as it should be up to 300 mL/30 min for casing cementing^41^. Also, the developed geopolymers possessed a fluid loss below 50 mL/30 min which is recommended for special cementing applications such as horizontal wells, and liner cementing^41^. The developed formulations provided this behavior without using a special fluid loss control additive.

### Sedimentation

**Figure 10:**
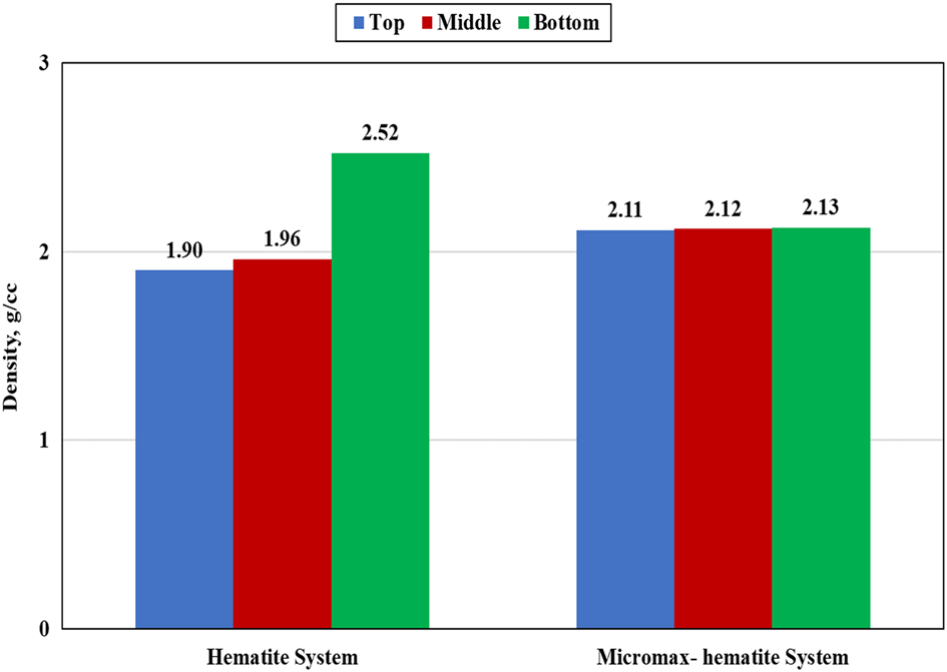
Effect of weighting agent on the density of the cylindrical geopolymer samples.

**Figure 11:**
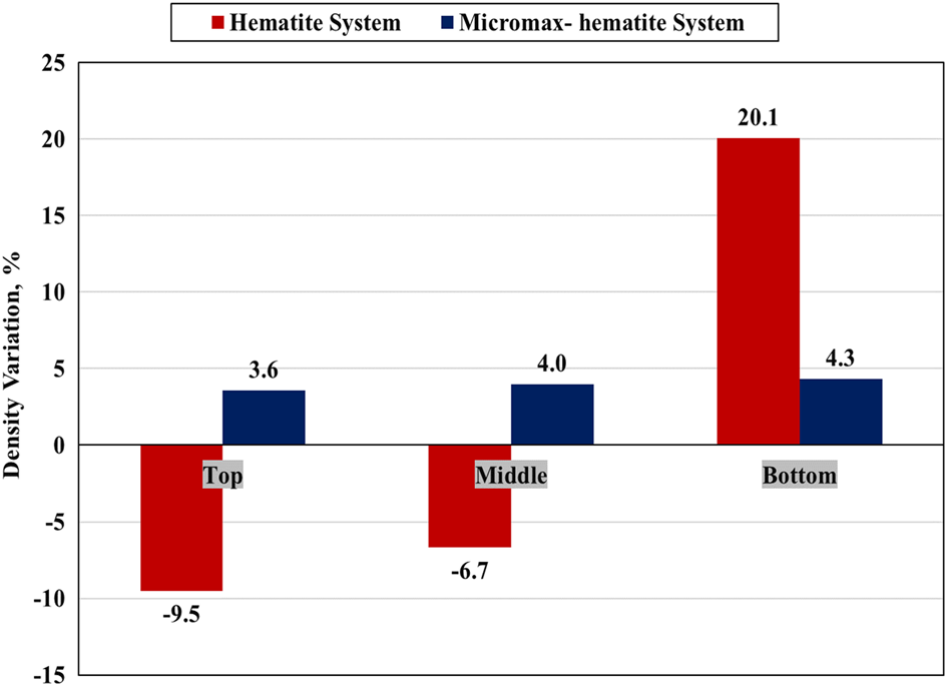
Density variation of the cylindrical geopolymer samples.

After curing at 292℉ and 3000 psi for 24 h using an HPHT curing chamber. Figures 10 and 11 show the density and density variation of different sections along the vertical orientation of the geopolymer samples. The sample heterogeneity can be expressed by the degree of density variation (DV) in such a way that the cement system becomes more homogeneous by decreasing the DV between the cement section and the slurry density. The particles sedimentation relies on size distribution, shape, density, and fluid viscosity through which particles are moving. The results confirmed that the hematite system had a high sedimentation tendency with a density difference of 32.6% between the bottom and top sections while the Micromax system had only 0.95%.

The hematite SG is larger than that of Micromax which boosts sedimentation. In addition, particle size distribution affects sedimentation tendency. The data, as shown in Table 1 and Fig. 1, confirmed that the D_10_, D_50_, and D_90_ for hematite were larger than those for Micromax. Generally, small particles have a higher surface area to mass ratio, so frictional drag slows their settling rate more than it does for larger particles. The harsh pressure and temperature conditions could affect the slurry viscosity resulting in a reduction in suspension capacity. Hematite has irregularly shaped particles, whereas Micromax particles are more approximately spherical in shape as shown by SEM in Fig. 4. Round particle settles out more quickly than flat, angular, or irregularly shaped particles because friction reduces for rounded particles. The overall sedimentation behavior is controlled by the interaction of the abovementioned components that made SG and PSD prevailing factors and shape a minor element. The free fluid of the geopolymer slurries was almost zero (traces). For gas migration control, the slurry should have a fluid loss less than 50 mL/30 min and zero free fluid, and these requirements are fulfilled in the developed geopolymer slurries^41^.

### Strength

**Figure 12:**
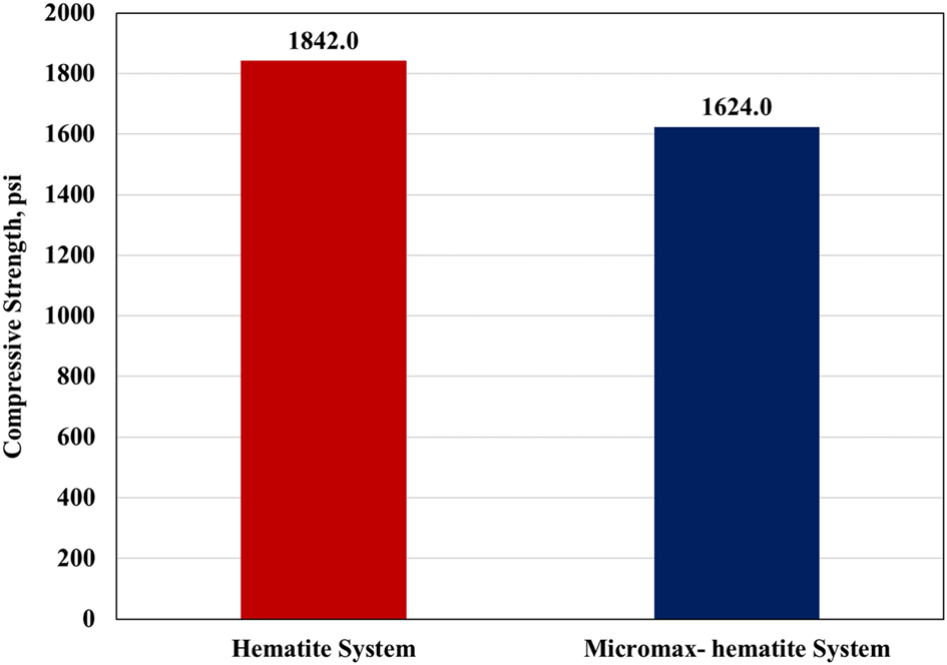
Compressive strength of both systems using scratch test.

**Figure 13:**
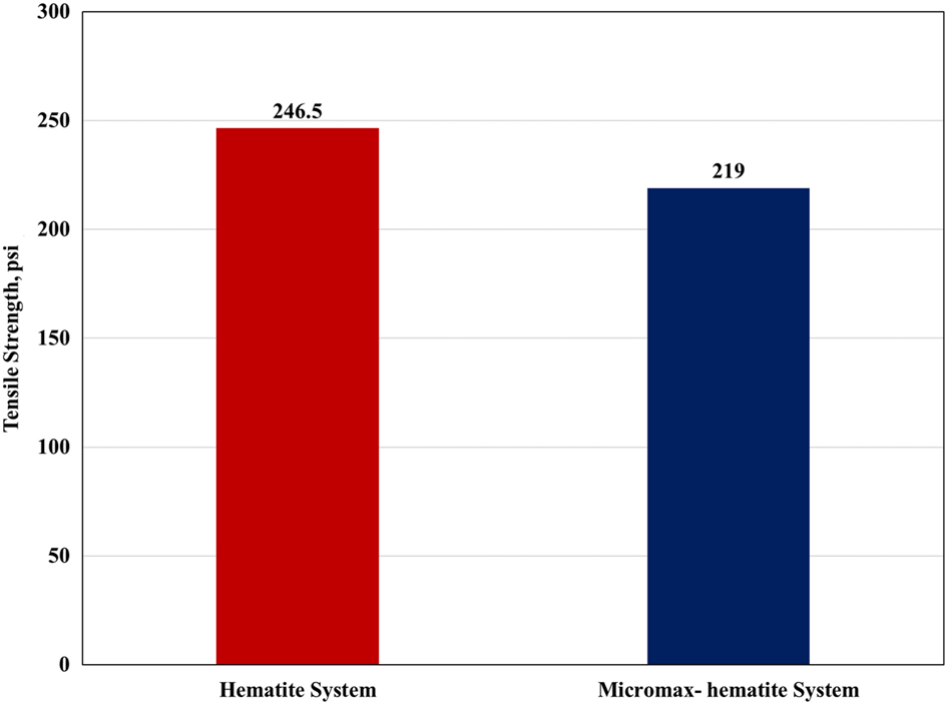
Tensile strength of both systems using the Brazilian Test.

The hematite-based system 24 h-compressive strength is larger than that of the mixed system by 13.4% and its tensile strength is greater by 12.5% as presented in Figs. 12 and 13. However, the mixed based geopolymer possessed a 24 h-compressive strength of 1624 psi which is more than three times the required value (500 psi) to resume drilling. Before resuming drilling or completion operations, the cement must harden and gain enough strength to support casing and seal off fluid movement behind casing. It is difficult to pinpoint the precise compressive strength required before drilling through the casing shoe, however, a minimum of 500 psi is recommended in the field practice^40^. Maximizing compressive strength has been a traditional approach in the past. The "more is better" philosophy may make this appear reasonable in the short term. However, in long term during well life, load conditions may take place where large strength may conflict with effective sealing and integrity support^40^.

The developed geopolymer formulations possessed a 24 h-compressive strength of at least 1,624 psi and a tensile strength of at least 219 psi which are greater than the required values for different purposes in the literature and some reported values in high density cementing programs in the Middle East. Farris^42^ experimentally showed that a tensile strength of only a few psi was adequate to support the casing weight under laboratory conditions; however, some considerations should be taken in case of dynamic loading imposed by drillstring rotation while subsequent drilling. Clark^43^ investigated the required strength to stop major fluid movement behind casing. The author concluded that tensile strength as low as 40 psi is accepted, with maximum bonding reached at roughly 100 psi. Another work mentioned that 8 psi tensile strength, or around 100 psi compressive strength is sufficient to support casing. There is harmony between the compressive strength and tensile strength results in such a way that the higher in compressive strength is also higher in tensile strength. Mitchell *et al*.^40^ mentioned that the ratio of compressive to tensile strength is approximately 8:1 to 12:1 for most cements and the study results (7.5:1) were so close to 8:1 ratio.

### Elastic properties and permeability

Cement sheath flexibility is significant where cement is under high stresses, e.g., steam injection, geothermal, and hydraulic fracturing. Flexible cements with a low Young’s modulus and a high Poisson’s ratio provide long-term well integrity. Set cement should have a lower YM than the surrounding formations. Small YM set cement may be preferable for unconsolidated strata than high YM set cement^41^. The proposed geopolymer systems possessed a YM of around 5.1 GPa and a PR in the range of 0.22–0.26 as listed in Table 5. The developed geopolymers are more flexible than Class G cement in terms of YM and PR as presented in Table 5. Moreover, they possessed lower YM than shale and consolidated formations and thus can be used adjacent to these formations as stated earlier by Liu^41^. Normally, more flexible cement, this with smaller YM and larger tensile strength, with enough compressive strength, performs well while simulating cement stresses. Permeability was measured for the two heavy weight geopolymer systems using Nitrogen. The liquid permeability was estimated by getting the y-axis intercept. The permeability was 0.015 and 0.009 md for the hematite and mixed geopolymer systems, respectively.

**Table 5:** YM and PR for some common materials against the developed geopolymers.

Material	YM (GPa)	PR (Dimensionless)
Hematite based geopolymer	5.18	0.26
Mixed based geopolymer	5.17	0.22
Extended cement systems	2.27	0.17
Flexible cement systems	1.99	0.22
Class G cement	11.03	0.17

## Conclusions

Two high density geopolymer systems using hematite alone and a mixture of hematite and Micromax were compared. The assessment included rheology (PV, YP, and GS), fluid loss, strength, permeability, and elastic properties. This study investigated the sedimentation problem associated with hematite as a weighting agent and introduced Micromax addition as a solution to mitigate this problem in geopolymers. The findings of this work can be summed up as follows:

- Replacing 50% of hematite with Micromax solved the sedimentation issue associated with the hematite-based geopolymer system.
- The Micromax-hematite weighted geopolymer had a density difference of 0.94% between top and bottom sections as compared to 32.6% for the hematite-based system.
- A proposed mixture of a retarder, a retarder intensifier and a superplasticizer was introduced that increased the thickening time of the mixed system by almost 3 times.
- The mixed geopolymer had lower fluid loss without using any fluid loss control additive that made it a good candidate for cementing applications such as horizontal drilling, gas migration risk, and casing cementing that require fluid loss less than 50 mL/30 min.
- At downhole temperatures, both systems followed Bingham plastic fluid model and the mixed system decreased the plastic viscosity by 44.8% and possessed a yield point close to the hematite-based system.
- The two formulations had close values in terms of strength, elastic properties, and permeability which fall within the accepted ranges of the industry.

## Data Availability

No external data was used for this research. All the generated experimental data are included in this manuscript.
